# Mechanism by Which Karrikin Priming Promotes Seed Germination of *Isodon serra* Under Heat Stress: Insights from Integrated Transcriptomic and Metabolomic Analyses

**DOI:** 10.3390/biology15141173

**Published:** 2026-07-16

**Authors:** Zijie Shen, Jinpeng Wei, Zhaoqi Zeng, Jie Wang, Qi Zhang, Jingfang Shi, Xiumei Li, Mengbing Li, Feili Zhou, Bingxian Chen, Aixia Zhang

**Affiliations:** 1 Guangdong Key Laboratory for Crop Germplasm Resources Preservation and Utilization, Agro-Biological Gene Research Center, Guangdong Academy of Agricultural Sciences, Guangzhou 510640, China; b02pnju2w3@163.com (Z.S.);; 2 Faculty of Science and Technology, Beijing Normal-Hong Kong Baptist University, Zhuhai 519087, China; 3 College of Agriculture and Biology, Zhongkai University of Agriculture and Engineering, Guangzhou 510550, China

**Keywords:** *Isodon serra*, heat stress, karrikin priming, seed germination, glutathione metabolism, antioxidant defense, multispectral phenotyping

## Abstract

*Isodon serra* is a medicinal plant distributed in the Lingnan region of China. However, *I. serra* seeds are sensitive to heat, limiting germination in Lingnan’s climate. Seed priming technology provides an effective means to address this problem. Meanwhile, karrikin (KAR) enhances plant growth and stress tolerance, but its mechanism in *I. serra* remains unclear. Therefore, in this study, different KAR concentrations were selected for priming to examine the phenotypic and physiological responses, and multi-omics analysis was used to explore the molecular mechanism of promoted germination under heat stress. The optimal concentration of 0.05 µmol/L KAR raised the seed germination rate under heat stress. This priming treatment reduced membrane damage, indicated by lower malondialdehyde (MDA) levels and relative electrical conductivity (REC) and oxidative stress, by regulating antioxidant enzymes and increasing glutathione (GSH) content. Multispectral phenotyping revealed distinct spectral features at 880 nm and 970 nm in primed seeds, correlating with enhanced vigor. Integrated multi-omics analysis demonstrated that the alleviation of heat stress was primarily mediated through the glutathione metabolism pathway, with key roles played by genes such as cluster-23509.3, cluster-15076.0, and cluster-22471.0. In summary, KAR priming enhances osmotic adjustment, improves reactive oxygen species (ROS)-scavenging enzyme activities, and regulates glutathione metabolism to mitigate heat stress in *I. serra*. These findings provide valuable insights for optimizing the cultivation and management of *I. serra* under heat stress conditions.

## Simple Summary

*Isodon serra* is a medicinal herb widely cultivated in southern China. It produces a suite of naturally occurring compounds with antitumor, anti-inflammatory, and hepatoprotective properties. A major constraint to its cultivation is that high temperatures severely reduce seed germination rates when sown in hot seasons. Here, we report that karrikin—a class of small signaling molecules derived from plant combustion smoke—effectively facilitates seed germination under heat stress. Notably, extremely low concentrations of karrikin were sufficient to significantly improve the germination of heat-stressed seeds. Parallel transcriptomic and metabolomic analyses were conducted to explore the underlying mechanism. Karrikin priming mainly strengthened antioxidant defense systems, and the glutathione pathway, which mitigates heat-induced oxidative damage, was identified as central to this response. Our results demonstrate that karrikin at ultra-low doses represents an effective seed treatment to enhance thermotolerance during germination while clarifying its molecular mode of action. These findings provide a simple and practical strategy for the stable propagation of this valuable medicinal herb under increasingly warm growing conditions.

## 1. Introduction

*Isodon serra* (family Lamiaceae) is a medicinal plant widely cultivated in southern China. Its main clinical value comes from ent-kaurane diterpenoids such as oridonin and ponicidin, which show antitumor, anti-inflammatory, and hepatoprotective activities [1]. Demand for the raw material has risen in recent years and put pressure on traditional field cultivation. The sowing season in southern China, however, falls during the hottest part of the year, and the germination of *I. serra* seeds declines sharply at these temperatures; such heat sensitivity occurs widely in Lamiaceae [2]. Because wild resources continue to dwindle, raw material quality now relies heavily on artificial propagation, which makes germination stability an increasingly practical concern [3]. This situation is unlikely to ease, since summer heat events in southern China are expected to grow more intense and frequent over the coming decades, shortening the usable nursery window [4]. At supra-optimal temperatures, imbibing seeds are damaged on several levels. Membrane lipid fluidity changes and destabilizes the membrane system, protein folding and translational homeostasis are disturbed, and ROS accumulate faster than they are cleared, so oxidative damage builds up [5,6]. Measures that keep seeds germinating under heat are therefore valuable both for understanding early stress adaptation mechanisms and for supporting the reliable propagation of high-value medicinal plants.

Seed priming refers to the controlled pre-imbibition of seeds in water or in solutions of active compounds. It has long been used to make germination more uniform and to improve stress tolerance in many crops [7,8,9]. Hormonal priming builds on this approach by applying endogenous or exogenous plant hormones to readjust the seed’s internal hormonal balance, raising vigor while leaving seed structure largely intact [10]. The hormones used this way include auxin, abscisic acid, gibberellin, brassinosteroid, and cytokinin [11]; even at very low concentrations, they act as signaling molecules that steer germination and later growth [12]. In high-value medicinal plants, though, these conventional agents often have narrow effective ranges, are awkward to apply, and differ widely in efficacy between species [13]. Priming agents that work at low doses, suit many species, and are simple to use therefore remain a research priority. Karrikins fit this profile: these small-molecule signals from wildfire smoke mimic post-fire germination cues and switch on DNA repair enzymes and heat tolerance gene networks, connecting environmental stress to the plant’s adaptive responses [10].

KAR molecule joins a pyran ring to a five-membered butanolide ring, and butanolide is responsible for activity [14]. KAR act at an extremely low threshold, tolerate heat, and show no genotoxicity [15,16]. The best-characterized natural form, KAR_1_ (3-methyl-2H-furo[2,3-c]pyran-2-one), is active at nanomolar to sub-micromolar concentrations—well under the doses normally needed for Gibberellic acid (GA_3_), Ethylene (ETH), or spermidine (Spd)—and promotes germination in crops, horticultural plants, and medicinal species [17,18]. Results like these have motivated closer studies of how karrikins regulate germination under stress.

In model plants, the signaling route is now fairly well defined: KAR is perceived by the receptor KAR_2_, which recruits MAX2 to mark SMAX1 and related SMXL repressors for degradation, lifting their block on downstream transcription [17,19,20]. KAR_2_ is related to the strigolactone receptor but controls a separate set of responses, including germination, photomorphogenesis, and the sensing of water status [21,22]. Applied karrikin can also improve tolerance to drought, salinity, and heavy metals, with reported effects on antioxidant capacity, osmolyte accumulation, and the abscisic acid/gibberellin (ABA/GA) balance [23]. In wheat, karrikin-active smoke fractions remodel flavonoid biosynthesis under salt stress and adjust both primary and secondary metabolism [24]. Germination under heat is a harder case. High imbibition temperatures suppress ribosome biogenesis and interfere with the selective translation of stored mRNAs that radicle emergence depends on [25,26], so a seed must have membrane stability, a working translational apparatus, and redox balance to germinate at high temperature [27]. These are the most likely points at which KAR keeps germination going above the optimal temperature. How it does so under heat stress is still unclear, and the question is even more acute in non-model medicinal plants, whose complex secondary metabolism makes borrowing conclusions from crop models unreliable.

Therefore, the present study aimed to examine how KAR priming improves *I. serra* seed germination under heat stress at the physiological, transcriptomic, and metabolomic levels, thereby revealing the mechanisms of seed thermotolerance and the molecular basis of KAR-promoted germination. This is also the first study to investigate KAR priming for promoting *I. serra* seed germination under heat stress. The findings confirm that KAR priming can effectively regulate *I. serra* seed germination under heat stress and enhance plant stress resistance.

## 2. Materials and Methods

### 2.1. Plant Material and Reagents

The *I. serra* seeds used in this study were obtained from long-term stocks maintained in a laboratory seed bank. After harvest, seeds were screened, placed in sealed bags containing desiccant, vacuum-sealed, and stored in a low-temperature, low-humidity cabinet at 5 °C and 50% relative humidity.

Karrikin was purchased from Hubei Shengguo Biotechnology Co., Ltd., Wuhan, China; spermidine (Spd) was purchased from Shanghai Macklin Biochemical Co., Ltd., Shanghai, China; gibberellin (GA_3_) and ethephon (ETH) were purchased from Hubei Lanze Biotechnology Co., Ltd., Wuhan, China. Assay kits for malondialdehyde (MDA), superoxide dismutase (SOD, WST-8 method), catalase (CAT, ammonium molybdate colorimetric method), peroxidase (POD), and reduced glutathione (GSH) were all purchased from Suzhou Comin Biotechnology Co., Ltd., Suzhou, China. REC measurement selected LeiCi DDS-307A, from Shanghai INESA Scientific Instrument Co., Ltd., Shanghai, China. All chemical reagents were of analytical grade. All assays were performed according to the manufacturer’s instructions.

### 2.2. Experimental Design

#### 2.2.1. Determination of Optimal Germination Temperature and Heat Stress Temperature

Healthy *I. serra* seeds were germinated under four temperature gradients of 20, 25, 30, and 35 °C (germination conditions described in Section 2.2.3), with 20 °C serving as the control. Each temperature treatment included three biological replicates, with 100 plump, uniformly sized *I. serra* seeds placed in a Petri dish supplied with an equal amount of distilled water in each replicate. Germination indices were measured at each temperature. A comprehensive evaluation of seed thermotolerance during germination was used to determine the optimal germination temperature and the optimal heat stress temperature.

#### 2.2.2. Screening of Effective Priming Agents

To screen for priming agents capable of alleviating heat stress, the optimal priming concentrations for different agents previously determined in *I. serra* heat stress germination experiments by the laboratory (in press) were used as reference. *I. serra* seeds were primed with 17.5 µmol/L GA_3_, 0.05 µmol/L KAR, 1 µmol/L Spd, or 1 µmol/L ETH, with dry seeds (CK1) and water-primed seeds (CK2) serving as controls. Priming was performed by mixing seeds with the priming solution at approximately 1:5 (*v*/*v*), incubating in the dark at 25 °C for 12 h, three times with distilled water, and then re-drying at 28 °C until the initial seed mass was restored (gravimetric method). The primed seeds were then subjected to a 7-day germination test at 35 °C. Based on the integrated germination indices under heat stress, 0.05 µmol/L KAR was selected for the subsequent physiological and molecular mechanism studies.

#### 2.2.3. Germination Test and Determination of Germination Indices

Germination tests were performed using the paper bed method. Filter paper was laid at the bottom of a 90 mm Petri dish, 8 mL of distilled water was added, and 100 seeds were evenly arranged in each dish. Each dish served as one biological replicate (three replicates per treatment). Dishes were sealed with parafilm to prevent water evaporation and placed in a controlled-environment chamber under a 16 h light/8 h dark photoperiod, 60% relative humidity, and a temperature set according to each treatment. The position of each dish within the chamber was randomized. Distilled water was replenished daily as needed to keep the filter paper moist.

Germination was scored daily. Germination energy was recorded on day 3 after sowing; the germination rate and germination index were calculated on day 7. Ten typical, representative seedlings were randomly selected from each replicate, and their radicle lengths were measured with vernier calipers to calculate the vigor index. A seed was considered germinated when the radicle clearly penetrated the seed coat with a length ≥ 1 mm.

Germination energy (%) = (*Gt*/*T*) × 100, where *Gt* is the number of seeds germinated on day 3, and *T* is the total number of seeds in the dish.

Germination rate (%) = (*Gt*/*T*) × 100, where *Gt* is the total number of seeds germinated on day 7, and *T* is the total number of seeds in the dish.

Germination index = Σ(*Gt*/*Dt*), where *Dt* is the number of days of germination; the germination index is calculated as the cumulative sum of the ratio of daily germinated seeds to the corresponding day.

Vigor index = Germination index × *S*, calculated as the product of the germination index and the seedling root length, where *S* represents the average hypocotyl length.

#### 2.2.4. Determination of Physiological and Biochemical Indicators

Sampling and measurement procedure: To evaluate the physiological response of *I. serra* seeds to KAR priming under high temperatures, we compared KAR-treated seeds (primed with 0.05 µmol/L KAR, 35 °C) against two controls, CK1 (hydroprimed, 25 °C) and CK2 (hydroprimed, 35 °C), each with three biological replicates. Given that preliminary tests showed >50% germination for all groups within 48–60 h, a sampling timepoint of 54 h was chosen to conduct physiological, multispectral, and multi-omics analyses.

*I. serra* seeds imbibed for 54 h under each treatment were rapidly collected to measure five physiological indicators: malondialdehyde (MDA) content, relative electrical conductivity (REC), superoxide dismutase (SOD) activity, catalase (CAT) activity, and reduced glutathione (GSH) content. Frozen samples were ground in liquid nitrogen and then processed following the instructions of the kits from Suzhou Comin Biotechnology Co., Ltd., Jiangsu, China. Sample homogenization, extraction buffers, centrifugation at 4 °C, and reaction temperatures all followed each kit’s standard protocol.

Specific measurement methods: Malondialdehyde (MDA) content was determined by the thiobarbituric acid (TBA) method, with absorbance read at 532 nm and 600 nm.

Catalase (CAT) activity was determined by the ammonium molybdate colorimetric method, monitoring H_2_O_2_ decomposition at 240 nm.

Superoxide dismutase (SOD) activity was determined by the WST-8 method, with absorbance read at 450 nm.

Reduced glutathione (GSH) content was determined by the 5,5′-dithio-bis (2-nitrobenzoic acid) (DTNB) reaction method, measured at 412 nm.

Relative electrical conductivity (REC) was measured by the standard soaking method: Approximately 0.1 g of frozen seeds was rinsed with distilled water and placed in a 50 mL centrifuge tube together with 30 mL of deionized water; the tube was soaked at 35 °C for 24 h, and the initial conductivity *T*1 was measured. The tube was then heated in a boiling water bath for 30 min and allowed to cool naturally to room temperature, after which the contents were thoroughly mixed, and the final conductivity *T*2 was measured [28].

(1) $$\mathrm{REC}=\frac{T1}{T2}\times 100\%$$

#### 2.2.5. Multispectral Imaging Analysis

On day 7 of imbibition, 10 seedlings were randomly selected from each of the three treatment groups—CK1 (hydroprimed, 25 °C), CK2 (hydroprimed, 35 °C), and KAR (primed with 0.05 µmol/L KAR, 35 °C)—and imaged with a VideometerLab 4 multispectral imaging system (Videometer A/S, Herlev, Denmark). Before sample acquisition, radiometric response, geometric alignment, and illumination were systematically calibrated using the manufacturer’s white, black, and dotted-white standard plates. Samples were placed inside an integrating sphere with a matte-white inner coating to ensure uniform diffuse illumination. LED flashes were used to sequentially expose each spectral band, yielding 2192 × 2192-pixel images (40 µm·pixel^−1^) with a 32-bit dynamic range; exposure time was automatically adjusted based on representative regions of interest to avoid overexposure while maintaining sufficient dynamic range. RGB images were simultaneously acquired by the same sensor. Image analysis was performed in VideometerLab 4™ (v3.14.9): individual-seed regions of interest (ROIs) were first extracted using the built-in binary large object (Blob) toolbox (one BLOB per seed), and images were transformed by normalized canonical discriminant analysis (nCDA). Morphological and color features—area, length, width, length-to-width ratio, circular compactness, BetaShape shape parameters, CIE Lab color-space parameters, saturation, and hue—were exported from the RGB images, and the mean reflectance spectra of seed pixels across the 19 bands were exported from the “statistic” module.

Although the instrument can acquire up to 19 monochromatic bands across the ultraviolet, visible, and near-infrared regions, only RGB images and six representative narrow-band images were retained for this study, namely 365 nm (ultraviolet, UV-A), 450/540/630 nm (visible blue, green, and red), and 880/970 nm (near-infrared). Each narrow-band image was z-score-normalized within the three sample groups, with intensity uniformly mapped to the range of −2 to +2 to enable direct comparison among the CK1, CK2, and KAR groups. Seed spectral–structural patterns were analyzed using the nCDA classification module built into VideometerLab; nCDA analyses were carried out using the candisc package in R (v4.4.1), visualizations were produced with ggplot2 (v3.3.3), and all multivariate results were visualized in parallel using the Transformation Builder module integrated in VideometerLab (v3.14) [29].

#### 2.2.6. Sampling and Analytical Procedures for Multi-Omics Sequencing

**Figure 1:**
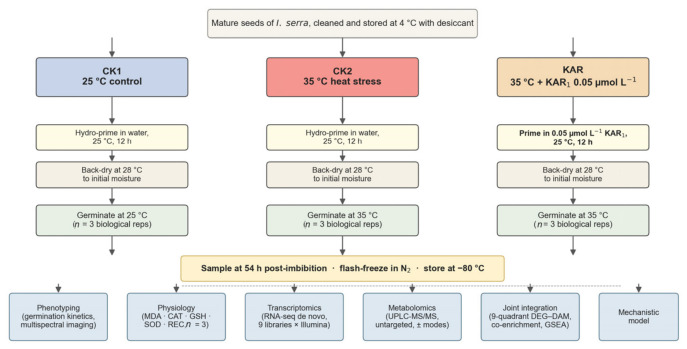
Experimental design and data collection workflow.

For the molecular mechanism experiments, three treatment groups were established—CK1 (hydroprimed, 25 °C), CK2 (hydroprimed, 35 °C), and KAR (primed with 0.05 µmol/L KAR, 35 °C). Each treatment included three biological replicates. Priming and re-drying procedures were as described above. Seeds were sampled 54 h after sowing, at which point approximately half of the KAR-primed seeds had begun to show radicle protrusion. Because sampling was performed at a uniform imbibition time rather than at matched developmental stages, the CK1, CK2, and KAR groups were not necessarily at fully synchronized developmental stages at the moment of sampling; this developmental asynchrony is acknowledged as a limitation in Section 4. Germination status was recorded at 54 h for each group. Within each biological replicate, an equal number of germinated seeds (including both protruded and non-protruded seeds) and non-germinated seeds were pooled for multi-omics sequencing (Figure 1).

#### 2.2.7. RNA Extraction, Library Construction, and Sequencing

Total RNA was extracted from frozen seed samples using the TRIzol method. Frozen samples were ground in liquid nitrogen into fine powder, transferred to pre-chilled 1.5 mL centrifuge tubes, and mixed with 1 mL of TRIzol reagent. After mixing and incubating at room temperature for 5 min, 200 µL of chloroform was added and incubated for a further 5 min at room temperature, followed by centrifugation at 17,000× *g* and 4 °C for 5 min. The upper aqueous phase was transferred to a new centrifuge tube, an equal volume of isopropanol was added, and the mixture was incubated at room temperature for 10 min before centrifugation at 17,000× *g* and 4 °C for 10 min. The pellet was washed twice with 1 mL of pre-chilled 75% ethanol (4 °C, 17,000× *g*, 10 min), air-dried at room temperature for 3–5 min, and dissolved in 20–50 µL of DEPC-treated RNase-free water.

RNA concentration and purity were measured with a NanoDrop spectrophotometer (Thermo Fisher Scientific, Waltham, MA, USA), and RNA integrity was assessed using an Agilent 2100 Bioanalyzer (Agilent Technologies, Santa Clara, CA, USA); only samples with an RNA integrity number (RIN) ≥ 7.0 were retained for library construction. Nine cDNA libraries (three biological replicates per treatment) were constructed and sequenced by Wuhan Metware Biotechnology Co., Ltd., Wuhan, China on the Illumina high-throughput sequencing platform, generating 150 bp paired-end reads.

#### 2.2.8. Transcriptome Assembly, Annotation, and Differential Expression Analysis

Raw sequencing data were processed using fastp (v0.23.2) to remove adapter sequences and filter low-quality reads, with Q20, Q30, and GC content used as quality metrics. High-quality reads from the nine libraries were assembled de novo using Trinity (v2.13.2), and transcripts were further clustered into non-redundant unigenes using Corset (v1.09). Assembly quality was assessed by unigene completeness and length distribution. Coding sequences (CDSs) were predicted using TransDecoder (v5.3.0). Unigenes were annotated against seven public databases (NR, Swiss-Prot, TrEMBL, KEGG, GO, KOG/COG, and Pfam) using Diamond (v2.0.9) in blastx mode with an E-value threshold of 1 × 10^−5^. High-quality reads from each library were aligned to the assembled transcriptome using Bowtie2 (v2.4.5), and transcript abundance was estimated with Rsem (v1.3.1). Expression levels were reported as fragments per kilobase of transcript per million mapped reads (FPKM); raw expected counts were used for differential expression analysis.

Differentially expressed genes (DEGs) were identified using DESeq2 (v1.22.2) based on raw expected counts, with thresholds of | Log_2_FC | ≥ 1 and *p* < 0.05. Two contrasts were analyzed: CK2 vs. CK1 and KAR vs. CK2. A Gene Ontology (GO) enrichment analysis and KEGG pathway over-representation analysis (ORA) of DEGs were performed using hypergeometric tests with *p* < 0.05. For the KAR vs. CK2 contrast, gene set enrichment analysis (GSEA) was performed using log_2_ fold change as the ranking metric, with KEGG pathway gene sets from the *Isodon serra* unigene collection as the reference set.

#### 2.2.9. Untargeted Metabolomic Profiling and Data Processing

(1) Sample preparation

Untargeted metabolomic analysis was performed by Wuhan Metware Biotechnology Co., Ltd. Frozen samples were freeze-dried using a Scientz-100F vacuum freeze-dryer and then ground into fine powder using a Retsch MM400 ball mill (30 Hz, 1.5 min). Approximately 50 mg of powder was weighed and extracted with 1200 µL of 70% aqueous methanol (containing an internal standard) pre-chilled to −20 °C; the sample was vortexed for 30 s every 30 min for a total of six cycles and then centrifuged at 14,000× *g* and 4 °C for 3 min. The supernatant was filtered through a 0.22 µm membrane and analyzed by UPLC-MS/MS.

(2) Chromatographic conditions

Samples were analyzed on a Waters ACQUITY UPLC system coupled to an AB SCIEX quadrupole–time-of-flight mass spectrometer. Chromatographic separation was performed on a Waters ACQUITY Premier HSS T3 column (1.8 µm, 2.1 mm × 100 mm; column temperature 40 °C). Mobile phase A was 0.1% formic acid in water, and mobile phase B was 0.1% formic acid in acetonitrile. The elution gradient was as follows: B was raised from 5% to 20% within 2 min, from 20% to 60% within 3 min, and from 60% to 99% within 1 min; held at 99% B for 1.5 min; returned to 5% B within 0.1 min; and finally held at 5% B for 2.4 min. The flow rate was 0.4 mL·min^−1^, and the injection volume was 4 µL. Positive- and negative-mode electrospray ionization (ESI) used the same elution gradient.

(3) Mass spectrometry conditions

Mass spectral data were acquired in information-dependent acquisition (IDA) mode using Analyst TF 1.7.1 software (Sciex, Concord, ON, Canada). Ion source parameters were as follows: ion source gas 1 (GAS1) at 50 psi, ion source gas 2 (GAS2) at 50 psi, curtain gas at 25 psi, ion source temperature at 550 °C, declustering potential at ± 60 V, and ion spray voltage at 5000 V in positive mode and −4000 V in negative mode. TOF-MS first-stage scans covered the *m*/*z* range 50–1000 Da with an accumulation time of 200 ms and dynamic background subtraction enabled; second-stage product ion scans covered the *m*/*z* range 25–1000 Da with an accumulation time of 40 ms, collision energy of ± 30 V, collision energy spread of 15, mass accuracy of 50 ppm, and a maximum of 18 candidate precursor ions selected per acquisition cycle.

(4) Data processing and DAM screening

To ensure analytical consistency, quality control (QC) samples were created by pooling equal aliquots of all biological specimens. These QC samples were inserted into the analytical sequence at regular intervals (after every 10 test samples) to track instrumental performance over time, and the order of sample injection was fully randomized. Metabolite identification was achieved by comparing experimental mass spectra against both the Metware proprietary database (MWDB) and publicly available metabolite repositories. Group-wise comparisons of metabolite profiles were conducted using orthogonal partial least-squares discriminant analysis (OPLS-DA) implemented in the R package MetaboAnalystR. Prior to model construction, raw data underwent log_2_ transformation and mean centering, and 200 permutation tests were executed to evaluate potential overfitting. Differentially accumulated metabolites (DAMs) were defined based on two criteria: a variable importance in projection (VIP) score > 1 derived from the validated OPLS-DA model, and a Benjamini–Hochberg adjusted two-tailed Student’s *t*-test with a significance threshold of *p* < 0.05. Hierarchical clustering analysis and Pearson correlation analysis were carried out using the R package ComplexHeatmap (v 2.22.0).

#### 2.2.10. Integrated Transcriptome–Metabolome Analysis

To identify pathways that changed coordinately at both the transcriptional and metabolic levels, DEGs and DAMs from the same contrast were jointly mapped to KEGG pathways, and shared pathways significantly enriched in both omics layers were identified by comparing the enrichment results. To further characterize the direction and significance of change for each gene–metabolite pair, a nine-quadrant analysis was performed. All detected gene–metabolite pairs were included; for each pair, the log_2_ fold change and adjusted *p* value of the gene were plotted against the corresponding values of the metabolite. Symmetric thresholds of | Log_2_FC | ≥ 1 and *p* < 0.05 were then applied to both omics layers, dividing all pairs into three categories: coordinated changes (quadrants 1, 3, 7, and 9), discordant or single-layer significant changes (quadrants 2, 4, 6, and 8), and the central non-significant region (quadrant 5). All analyses and plots were generated using the Metware Cloud, a free online data analysis platform (https://cloud.metware.cn).

#### 2.2.11. RT-qPCR Analysis

First-strand cDNA was synthesized using the PrimeScript™ RT Reagent Kit with gDNA Eraser (TaKaRa Biotechnology (Dalian) Co., Ltd., Dalian, China) according to the manufacturer’s instructions.

The reverse transcription reaction was performed in a thermocycler under the following standard program: 25 °C for 12 min, 50 °C for 25 min, and 85 °C for 5 min. First-strand cDNA was synthesized from gDNA-free RNA; after the reaction, cDNA could be stored at −20 °C or diluted for immediate use in subsequent experiments. For long-term storage, cDNA was aliquoted and stored at −80 °C.

RT-qPCR validation was performed using the 2× RealStar Green Fast Mixture kit (GenStar, A301-10) on eight differentially expressed genes randomly selected from key enriched metabolic pathways. Three biological replicates were set for each *I. serra* sample, with three technical replicates per biological replicate. The 18S ribosomal RNA gene (figshare: 26827906) was used as the internal reference, and the 2^−ΔΔCt^ method was used to calculate the relative expression of target genes.

#### 2.2.12. Data Analysis

Phenotypic, physiological, and transcriptomic measurements were performed in three replicates, while metabolomic analyses used six replicates. Statistical analyses were performed in IBM SPSS Statistics 27. A one-way analysis of variance (ANOVA) was used to assess the physiological and biochemical effects of each treatment, and Fisher’s least significant difference (LSD) test was used for pairwise comparisons of means. A *p* value of less than 0.05 was considered statistically significant. Data are presented as means ± standard deviation (*n* = 3). Plots were generated using GraphPad Prism 9, R (v 4.1.3), Python (v 3.10), and Adobe Illustrator 2023. Correlation coefficient heatmaps and Pearson correlation-based distance measurements were generated using the Metware Cloud (https://cloud.metware.cn).

## 3. Results

### 3.1. KAR Priming Alleviates the Germination Inhibition of I. serra Seeds Under High Temperature

**Figure 2:**
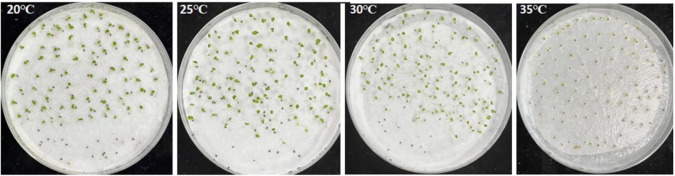
Germination of *I. serra* seeds under different temperatures.

**Figure 3:**
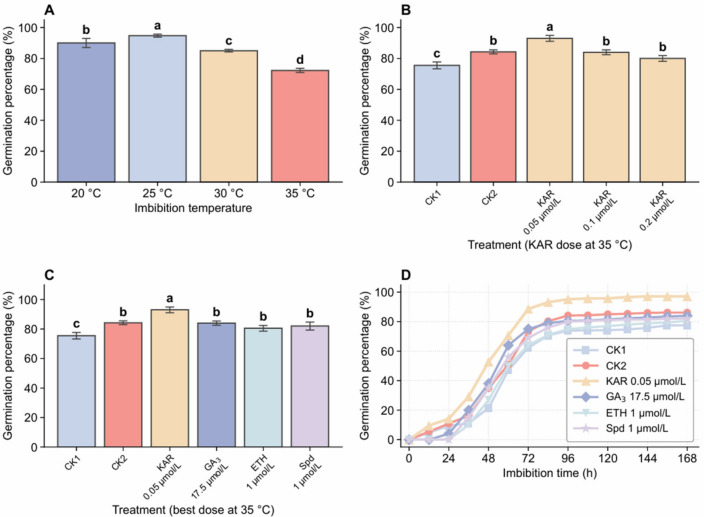
This figure shows 35 °C as the optimal heat stress temperature and 0.05 µmol/L KAR as the optimal priming concentration. Note: (**A**) Germination percentage under different imbibition temperatures (20, 25, 30 and 35 °C); (**B**) germination percentage under different KAR concentrations (0.05, 0.1 and 0.2 µmol/L) at 35 °C; (**C**) germination percentage under different priming agents at their optimal concentrations at 35 °C; (**D**) germination time-course (0–168 h) under the six treatments. Significance analysis was performed using Duncan’s multiple range test and Student’s *t*-test; different letters indicate significant differences (*p* < 0.05). Control group: CK1 (unprimed), CK2 (water-primed).

The present study first germinated healthy, uniformly sized seeds at 20, 25, 30, and 35 °C (Figure 2) and recorded the germination indices after a 7-day culture period (Figure 3A). The results showed that germination was the best at 25 °C—the germination rate reached 95%, the germination index was 38.89, and the vigor index was 86.8 (Figure 2). Under other temperatures, all indices declined to varying extents, with germination being the most strongly inhibited at 35 °C: the germination rate dropped to 72%, the germination index to 25, and the vigor index to 17. Radicle growth showed the same trend, with the mean radicle length reaching 2.23 cm at 25 °C but only 0.68 cm at 35 °C (Figure 2; Table A1). Based on these results, 35 °C was selected as the heat stress temperature for subsequent experiments and 25 °C as the optimal control temperature.

**Figure 4:**
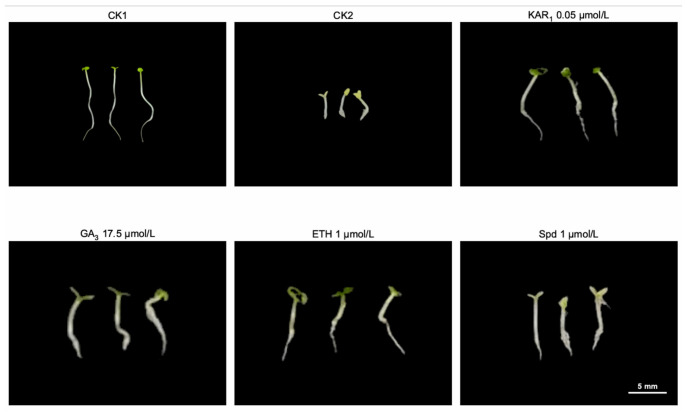
Effects of different priming agents at their optimal concentrations on growth of *I. serra* seedlings.

To screen for the most effective priming agent and optimal concentration to alleviate *I. serra* seed germination inhibition at 35 °C, a four-point concentration gradient experiment was conducted simultaneously at 35 °C for four priming agents (KAR, GA_3_, ETH, and Spd). Overall, the four priming agent treatments showed three distinct response patterns: KAR achieved the best effect at 0.05 µmol/L, with the effect declining when the concentration rose to 0.1 and 0.2 µmol/L—the germination rate was 94% at the lowest dose and 80% at the highest (Figure 3B; Table A2). The optimal doses of the other three priming agents were approximately 20–350 times (1–2.5 orders of magnitude) higher than that of KAR: 17.5 µmol/L for GA_3_, 1 µmol/L for ETH, and 1 µmol/L for Spd. At their respective optimal doses, the germination rates of the four priming treatment groups all reached 90–97%, all significantly higher than that of CK2. Among them, 17.5 µmol/L GA_3_ gave the single highest germination rate, while 0.05 µmol/L KAR achieved a comparable germination rate but at a working concentration approximately 350 times lower than that of GA_3_ (Figure 3C,D). For seedling phenotype, CK2 had the shortest root length among groups; among the priming treatments, only KAR-primed seedlings showed root lengths comparable to CK1, while CK1 seedlings exhibited smaller and more damaged cotyledons (Figure 4). Based on these results, 0.05 µmol/L KAR was selected as the priming condition for the subsequent multi-omics experiments.

### 3.2. KAR Priming Reduces Membrane Damage and Enhances Seed Antioxidant Enzyme Activity

To further investigate the physiological mechanism by which KAR priming acts on *I. serra* seeds under heat stress, five key physiological indicators—MDA content, REC, SOD activity, CAT activity, and GSH content—were rapidly measured in *I. serra* seeds after 54 h of imbibition. The aim was to characterize how KAR priming mitigates stress via effects on cell membrane integrity and antioxidant defense.

**Figure 5:**
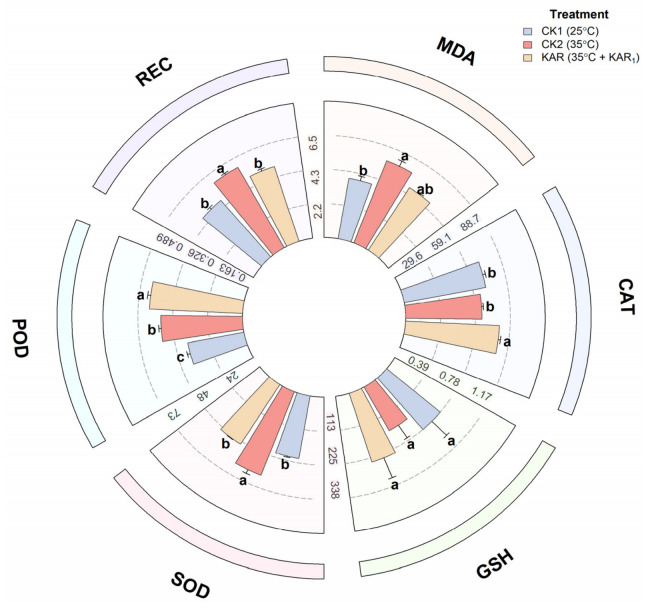
Effects of KAR priming on five physiological indicators of *I. serra* seeds under heat stress. Note: Significance analysis was performed using Duncan’s multiple range test and Student’s *t*-test; different letters indicate significant differences *(p* < 0.05). Control group: CK1 (hydroprimed, 25 °C), CK2 (hydroprimed, 35 °C). This sampling strategy was consistently applied across subsequent experimental groups. Bars represent CK1 (light blue), CK2 (red) and KAR (orange).

Compared with CK1, SOD activity in CK2 was significantly elevated by 41%, reflecting an emergency defense response after substantial reactive oxygen species accumulation; after KAR treatment, SOD activity returned to the CK1 level, with no notable difference between the two groups. CAT and POD activity showed a distinctly different pattern from SOD: CK2 treatment suppressed CAT and POD activity, whereas KAR treatment significantly elevated it, with values significantly higher than those of the other two groups. MDA content and REC were significantly elevated in CK2, indicating severe membrane damage, whereas no significant difference was observed between CK1 and KAR, indicating that KAR priming can effectively protect the integrity of the *I. serra* seed membrane system under heat stress. Finally, GSH did not differ significantly among the three groups, but the KAR-primed group had the highest content (Figure 5).

Overall, KAR priming both up-regulated some antioxidant enzyme activity and alleviated the excessive oxidative damage to the membrane system caused by heat stress, effectively regulating the homeostasis of the seed antioxidant and osmotic adjustment systems.

### 3.3. Multispectral Feature Analysis of I. serra Seeds After KAR Priming Under Heat Stress

**Figure 6:**
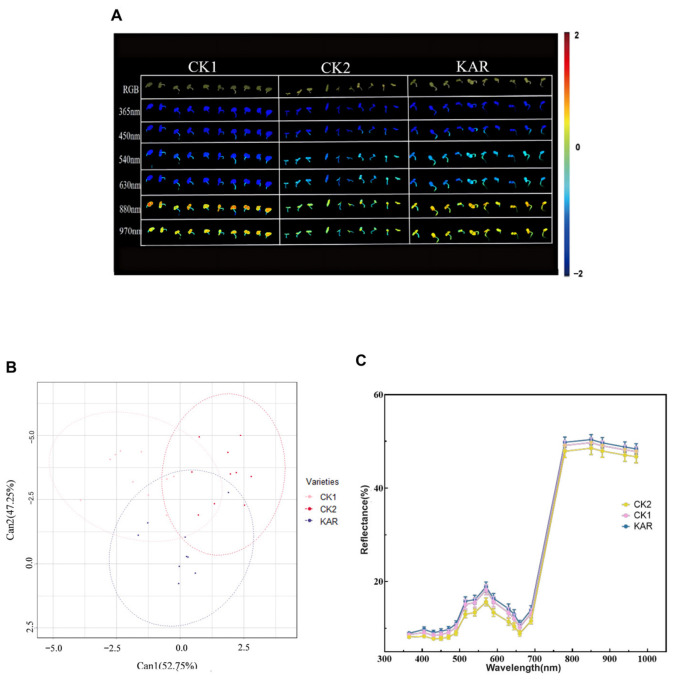
Multispectral imaging analysis of KAR priming-promoted germination of *I. serra* seeds under heat stress. Note: (**A**) Multispectral imaging of seedlings after 7 days of imbibition in three treatment groups at 35 °C; (**B**) nCDA score plot of seedlings after 7 days of imbibition in three treatment groups at 35 °C; (**C**) average spectral reflectance of seedlings after 7 days of imbibition in three treatment groups at 35 °C.

To further evaluate whether the germination phenotype of *I. serra* seeds after KAR priming differed at the spectral level, multispectral imaging analysis was performed on seedlings from the three treatment groups on day 7 of imbibition. Across the seven spectral views (the RGB image and six narrow-band reflectance images at 365, 450, 540, 630, 880, and 970 nm), the spectral features of KAR-primed seedlings were clearly closer to those of CK1, with a clear separation between CK2 and CK1. The separation was the most pronounced in the 880 nm and 970 nm near-infrared bands, in which CK1 showed the highest reflectance. The convergence of KAR group spectral features toward the CK1 pattern indicates that KAR-primed seeds exhibit an overall spectral phenotype similar to the unstressed control under heat stress, reflecting a more normalized state at the level of cellular physiology and metabolism (Figure 6A). The two-dimensional nCDA score plot also showed clear differences among the three treatment groups: within the confidence interval of each group, most samples were distinctly distributed, indicating a degree of phenotypic difference among the three treatments (Figure 6B). The average spectral reflectance line plot showed significant differences among the three groups in the 500–700 nm and 800–900 nm bands, suggesting that KAR priming may promote *I. serra* seed germination and seedling growth by modulating photosynthetic pigment synthesis and water content [29] (Figure 6C).

### 3.4. Transcriptomic Analysis

#### 3.4.1. Quality Control of Transcriptome Data

**Figure 7:**
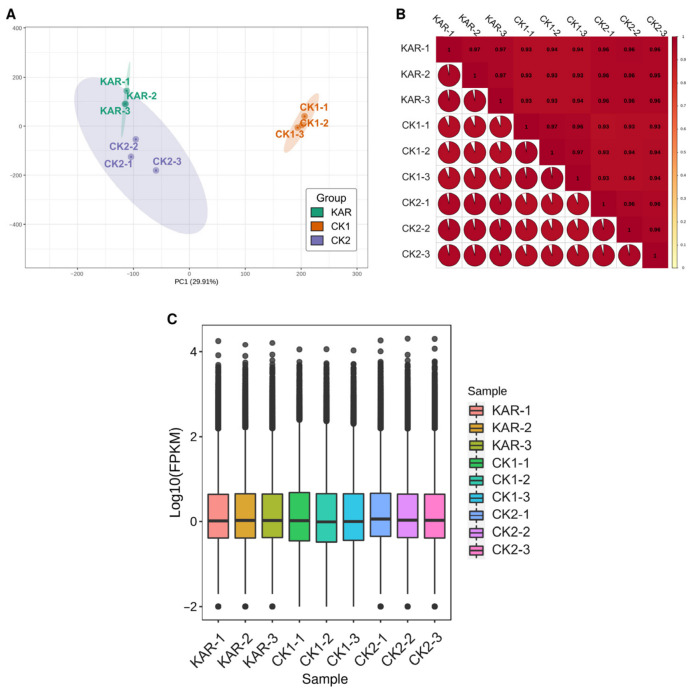
Quality control and sample correlation of transcriptome data. Note: (**A**) Principal component analysis (PCA) score plot of the nine samples; (**B**) Pearson correlation heatmap among the nine samples; (**C**) distribution of log10(FPKM) expression values for each sample.

To assess the reliability and biological reproducibility of the transcriptome data, total RNA was extracted from three replicates each of CK1, CK2, and KAR-treated seeds, and nine cDNA libraries were constructed and sequenced on the Illumina high-throughput platform. The nine libraries together generated 72.72 Gb of high-quality data, with effective data per sample ranging from 7.34 to 9.21 Gb (Table A3). Sequencing quality was stable across samples (Q20 ≥ 97.73%, Q30 ≥ 93.21%, error rate 0.01%, GC content 47.32–48.20%), and the de novo assembly of the high-quality reads produced 77,144 non-redundant unigenes. Quality control analyses including PCA, correlation heatmaps, and FPKM density distribution (Figure 7A–C) all indicated good sequencing results for the three sample groups, with no obvious outliers and high within-group consistency.

#### 3.4.2. Differential Expression Analysis of Common DEGs

**Figure 8:**
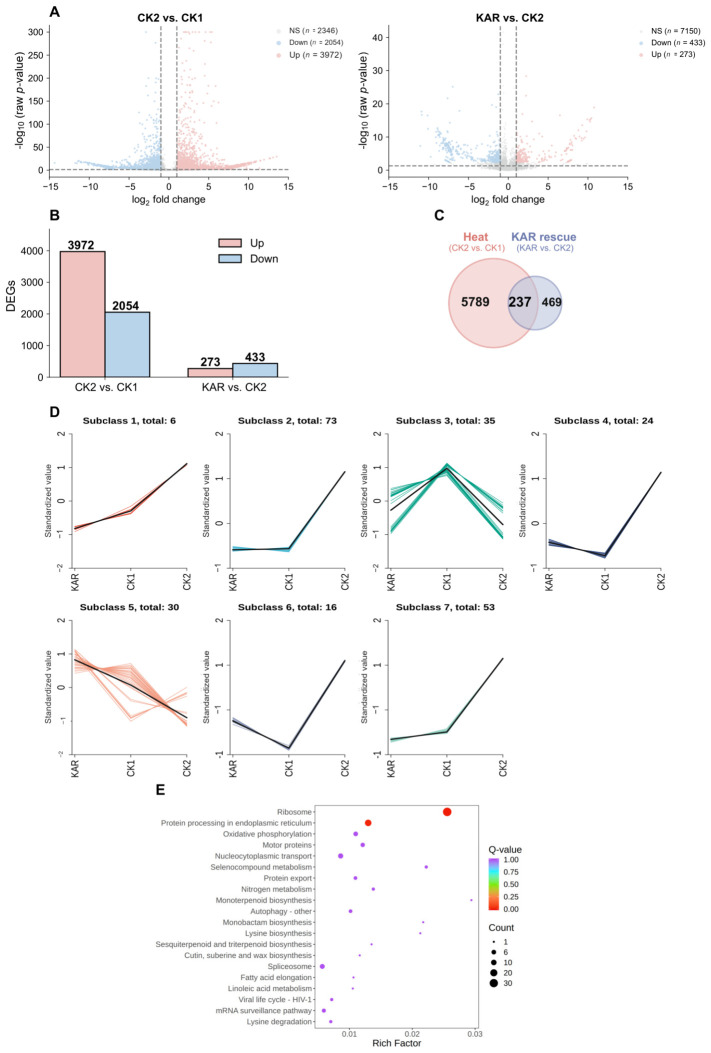
Analysis of 237 common DEGs in heat-stressed *I. serra* seeds. Note: (**A**) Volcano plots of DEGs in different comparison groups; red (up-regulated) and blue (down-regulated) points represent significant DEGs with |Log_2_FC| > 1 and *p* < 0.05, and gray points represent non-significant DEGs. (**B**) Bar chart of DEG counts in three sample groups. (**C**) Venn diagram of DEGs across three sample groups; different colors represent different comparison combinations. (**D**) K-means clustering analysis. (**E**) KEGG pathway enrichment of 237 common DEGs.

DEG analysis focused on two contrasts: CK2 vs. CK1 and KAR vs. CK2. In terms of CK2 vs. CK1, a total of 6026 differentially expressed genes were identified, including 3972 up-regulated and 2054 down-regulated ones; the volcano plot showed a dense distribution across the entire fold change range, consistent with a typical whole-transcriptome-level response. In contrast, the KAR vs. CK2 contrast was much narrower, with only 706 genes significantly differentially expressed (273 up-regulated and 433 down-regulated) (Figure 8A,B).

The Venn analysis between the two contrasts identified 237 common DEGs (Figure 8C). The K-means clustering of these 237 DEGs grouped them into seven expression pattern subclasses. Most genes were significantly up-regulated in CK2, and KAR treatment reversed the heat-induced expression of some of these genes while restoring others to levels close to CK1, indicating that KAR exerts a clear regulatory and restorative effect on heat-stress-responsive genes (Figure 8D).

Finally, the KEGG enrichment analysis of the common DEGs showed the highest enrichment in ribosomes, protein processing in the endoplasmic reticulum, and oxidative phosphorylation pathways, suggesting the marked activation of cellular protein synthesis and energy metabolism under heat stress. Among the enriched pathways, glutathione metabolism was also identified, indicating that heat stress triggered a strong oxidative stress response and that the plant alleviates heat-induced cellular damage by maintaining energy metabolism, protein repair, and antioxidant biosynthesis, thereby reducing oxidative stress and restoring proteostasis (Figure 8E).

#### 3.4.3. GSEA and *HSP* Gene Family Statistics

**Figure 9:**
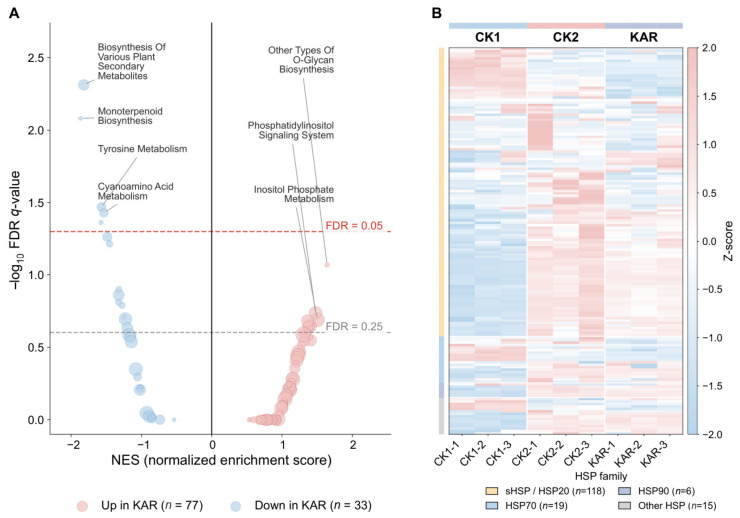
GSEA again identifies glutathione metabolism pathway as enriched, and *HSP* gene family expression differs among groups. Note: (**A**) GSEA pathway enrichment of all genes between KAR and CK2; NES values: positive indicates enrichment at top of ranked list (pathway genes up-regulated), and negative indicates enrichment at bottom (pathway genes down-regulated). (**B**) Heatmap of expression levels of *HSP* gene family members.

To reveal the regulatory features of KAR priming at the whole-transcriptome level, GSEA was performed on all genes from the KAR vs. CK2 contrast. With NES ≥ 1.5, 26 metabolic pathways were enriched in total, of which 5 reached FDR < 0.05 (Figure 9A). These five significant pathways were monoterpene biosynthesis, biosynthesis of plant secondary metabolites, tyrosine metabolism, cyanoamino acid metabolism, and benzoxazinoid biosynthesis, all located in the negative NES region, indicating that these pathways were coordinately down-regulated after KAR treatment.

In the positive NES region, the top enriched pathways included inositol phosphate metabolism (NES =1.50), phosphatidylinositol signaling system (NES = 1.48), glutathione metabolism (NES = 1.38), proteasome (NES = 1.37), and protein transport (NES = 1.34). Among these, glutathione metabolism was also enriched in the transcriptome KEGG analysis, and GSEA showed that the leading-edge core genes of this pathway were concentrated in the up-regulated region of the KAR treatment (Figure 9A). Overall, KAR regulation at the transcriptional level is targeted, modulating mainly the core pathways of this study rather than broadly amplifying the plant’s heat stress response.

Heat shock proteins (HSPs) are marker proteins for the heat stress response. A total of one hundred and fifty-eight *HSP* genes covering four families were identified from the annotation database: one hundred and eighteen *HSP20* family genes, nineteen *HSP70* family genes, six *HSP90* family genes, and fifteen other *HSP* genes. The expression of all these genes was z-score-normalized, and a gene expression heatmap of the nine samples was plotted (Figure 9B).

The *HSP20* and *HSP70* families showed the most pronounced response to KAR priming, with 97 and 16 down-regulated genes, respectively; the *HSP90* family showed a weaker but consistent downward trend, with 5 of its 6 genes being down-regulated. In addition, 63.3% of the 158 *HSP* genes showed expression levels under KAR priming that were much closer to the unstressed control than to those of the heat-stress-only group. Collectively, KAR priming can restore the expression of most heat-stress-induced *HSP* genes to control levels, indicating that during the sampling window of this experiment, KAR priming does not sustain or amplify the plant’s heat shock transcriptional response but instead effectively dampens the cellular stress signals that trigger *HSP* induction.

### 3.5. Untargeted Metabolomic Analysis

#### 3.5.1. Metabolite Structural Composition and Sequencing Model Quality Analysis

**Figure 10:**
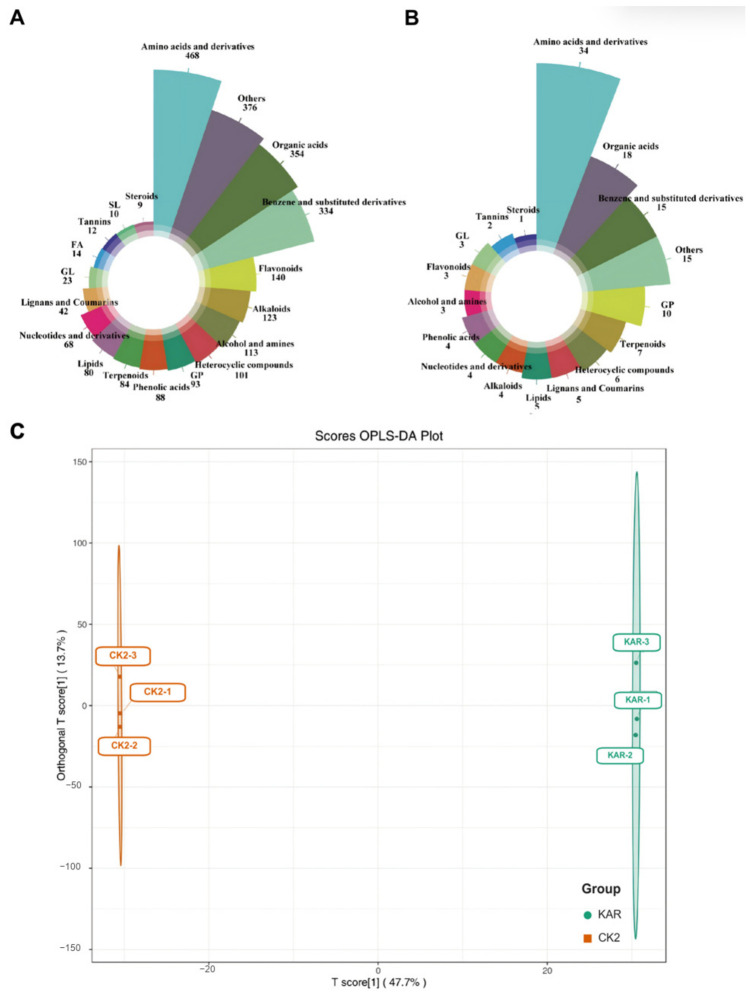
Chemical structure composition analysis of metabolites and OPLS-DA model statistics. Note: (**A**) Chemical-structure classification of all identified metabolites; (**B**) chemical-structure classification of the differentially accumulated metabolites (DAMs); (**C**) OPLS-DA score plot (KAR vs. CK2).

Untargeted metabolomic analysis was then performed on *I. serra* seeds from the different treatments. A total of 2434 metabolites were identified across all samples, and the differential metabolites could be classified into 19 chemical categories (Figure 10A). Amino acids and their derivatives accounted for the highest proportion (19.23%), followed by other metabolites (15.44%) and organic acids and their derivatives (14.54%). The identified metabolites covered the chemical classes commonly found in plants, including flavonoids, lipids, amino acids and their derivatives, organic acids, phenolic acids, terpenoids, and alkaloids. This profile is consistent with the secondary metabolic diversity expected from Lamiaceae medicinal plants.

Further chemical structure classification of the common DAMs likewise placed them into 19 categories. Amino acids and their derivatives still accounted for the largest share (24.46%), followed by phenylpropanoids and polyketides (14.54%) and organic acids and their derivatives (12.95%) (Figure 10B). These results indicate that under heat stress, KAR priming affects the biosynthesis of amino acids and organic acids in *I. serra* seeds.

Next, supervised orthogonal partial least-squares discriminant analysis (OPLS-DA) was used to assess the effects of the different treatments on the metabolome. The results showed a clear separation of KAR vs. CK2 in the score space and validated the model through 200 permutation tests: all permuted Q^2^ intercepts were clearly lower than the observed values, and no single permutation produced predictive power exceeding that of the original model (Figure 10C).

#### 3.5.2. Analysis of Common DAMs

**Figure 11:**
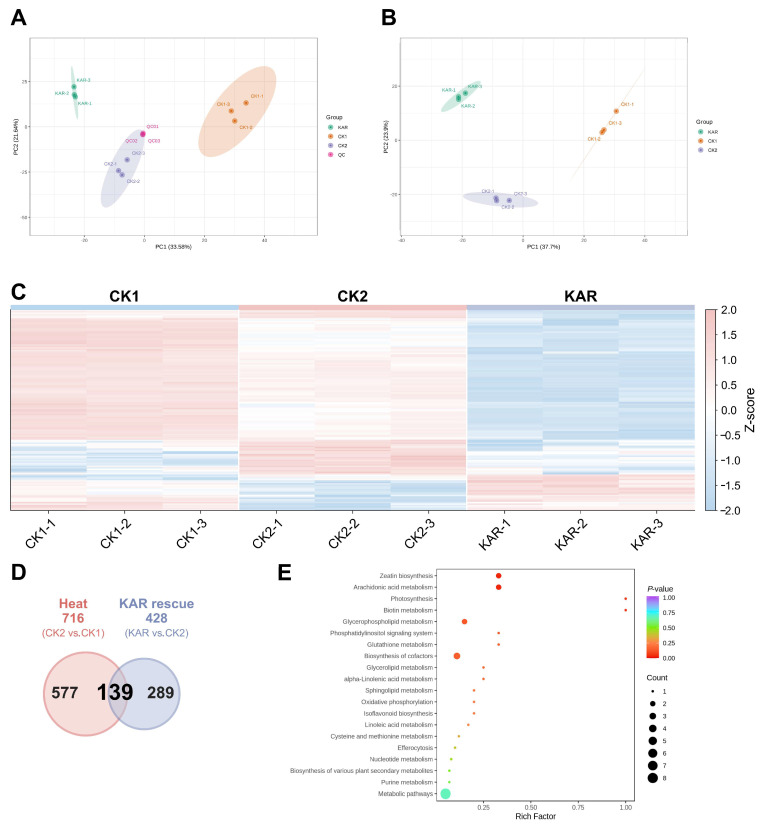
Screening and functional enrichment of common DAMs across different treatments. Note: (**A**) PCA score plot of metabolites in positive ion mode (including QC samples); (**B**) PCA score plot of metabolites in negative ion mode; (**C**) Correlation heatmap of common DAM expression among sample replicates; (**D**) Venn diagram of DAMs between comparison groups; (**E**) KEGG pathway enrichment of 139 common DAMs.

Principal component analysis showed that the three sample groups were clearly distinguishable in both ionization modes (Figure 11A,B). The first principal component reflected the sample differences induced by heat stress, while the second principal component reflected the restorative effect of KAR treatment, effectively separating the KAR group from CK2 samples.

The clustering heatmap of common DAMs visually illustrated the restoration pattern: the expression trends in most metabolites were opposite between CK1 and CK2; after KAR priming, the levels of these metabolites dropped sharply, becoming significantly lower than those of the control. This suggests that KAR priming promotes germination mainly by down-regulating the levels of certain metabolites in heat-stressed *I. serra* seeds (Figure 11C).

The statistics of between-group DAMs showed that the CK1 vs. CK2 comparison yielded 716 DAMs, and the KAR vs. CK2 comparison yielded 428 DAMs. The two groups shared 139 DAMs, all of which were affected by heat stress (Figure 11D).

Finally, KEGG enrichment analysis showed that the glutathione metabolism pathway remained significantly enriched, further confirming its importance in regulating *I. serra* seed germination under heat stress. Other enriched pathways included zeatin biosynthesis, arachidonic acid metabolism, and photosynthesis (Figure 11E).

### 3.6. Integrated Transcriptome–Metabolome Analysis of I. serra Seeds Under Heat Stress

#### 3.6.1. Nine-Quadrant and KEGG Co-Enrichment Analysis

**Figure 12:**
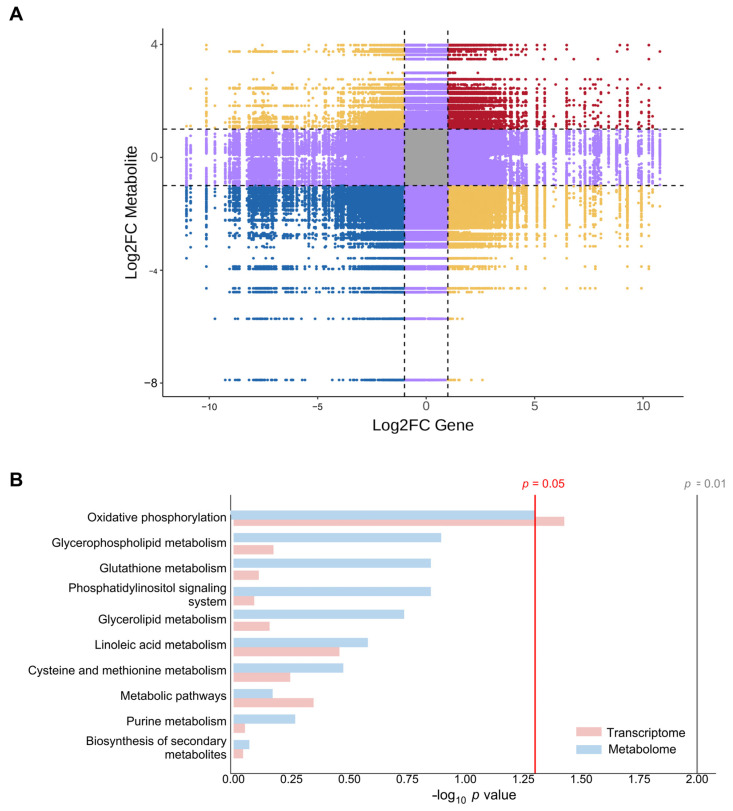
Multi-omics integrative analysis identifies glutathione metabolism pathway as central pathway. Note: (**A**) Nine-quadrant analysis of DEGs and DAMs. (**B**) KEGG co-enrichment analysis of DEGs and DAMs; blue columns represent KEGG enrichment of common DAMs, and red columns represent KEGG enrichment of common DEGs. In (**A**), point colors denote the nine quadrants of the DEG–DAM nine-quadrant analysis, with the gray central region indicating the non-significant quadrant.

The present study first analyzed the co-expression network between *I. serra* DEGs and DAMs to explore the relationship between gene expression and metabolic regulation. A nine-quadrant plot was used to characterize their correlation (Figure 12A). Each DEG–DAM pair was classified by its direction and significance of change: coordinated changes were assigned to quadrants 1, 3, 7, and 9; discordant or single-layer significant changes were assigned to quadrants 2, 4, 6, and 8; and the central non-significant region was assigned to quadrant 5. In CK2 vs. CK1, DEG–DAM pairs were densely distributed in all four diagonal quadrants, with quadrants 9 (co-up-regulated) and 1 (co-down-regulated) being particularly prominent, consistent with the expected coordinated response of transcription and metabolism to heat stress. The KAR vs. CK2 comparison showed a similar overall topology but with fewer pairs in each quadrant and a more concentrated distribution (Figure 12A).

Subsequent KEGG co-enrichment analysis identified 10 shared pathways enriched in both omics layers (Figure 12B). Of these pathways, only “oxidative phosphorylation” was significantly enriched at both the gene and metabolite levels. Other shared pathways with low *p* values on the gene side included glycerophospholipid metabolism, glutathione metabolism, phosphatidylinositol signaling, glycerolipid metabolism, linoleic acid metabolism, and cysteine–methionine metabolism. Integrating the transcriptome, metabolome, and GSEA results, the glutathione metabolism pathway was the only one consistently identified across all enrichment analyses. Together with the changes in physiological indicators, glutathione metabolism was selected as the key metabolic pathway for subsequent integrative analysis (Figure 12B).

#### 3.6.2. Identification of Hub Genes Related to Core Metabolites

**Figure 13:**
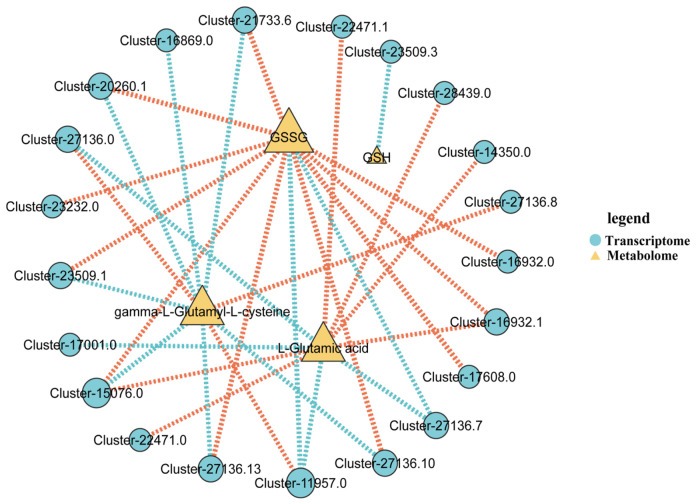
Correlation network analysis of glutathione metabolism pathway. Note: Blue circles represent DEGs, yellow triangles represent DAMs, red lines indicate positive correlations, and blue lines indicate negative correlations.

To identify gene–metabolite associations within the glutathione metabolism pathway, correlation analysis was further performed between the key DEGs and the core DAMs of this pathway. The results showed that GSSG was significantly positively correlated with most DEGs, suggesting positive regulation by the corresponding coding genes; in contrast, GSH was significantly negatively correlated only with cluster-23509.3, and γ-L-glutamyl-L-cysteine was significantly negatively correlated with most DEGs, suggesting negative regulation by these genes. Among them, two DEGs—cluster-15076.0 and cluster-11957.0—were significantly correlated with all three metabolites, indicating potentially important regulatory roles (Figure 13).

#### 3.6.3. Synthesis Regulation Network of Core Genes and Key Metabolites

**Figure 14:**
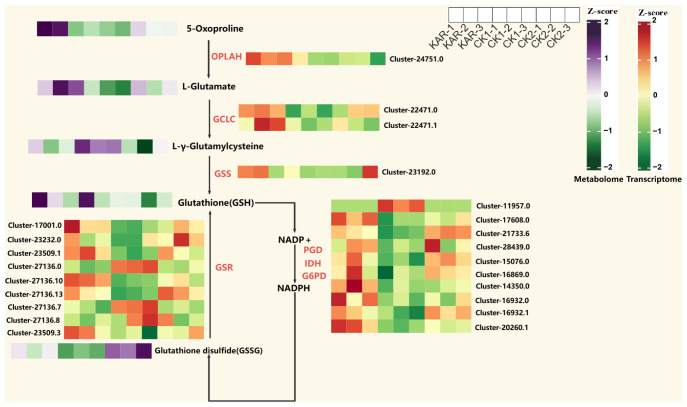
Co-expression regulatory network of glutathione metabolism pathway. Note: Red–green blocks indicate change in DEG expression (red, up-regulated; green, down-regulated); purple–green blocks indicate change in DAM levels (purple, up-regulated; green, down-regulated).

Based on the multi-omics integrative analysis, a gene–metabolite expression network heatmap of the glutathione metabolism pathway was constructed. A total of 22 DEGs were identified in the glutathione metabolism pathway, of which 17 were up-regulated under KAR priming (Figure 14), with key genes showing significantly differential regulation. The metabolic enzyme gene *OAT* (ornithine aminotransferase), which corresponds to the upstream metabolite 5-oxoproline, was significantly up-regulated in the KAR-primed group, suggesting that this metabolic step may be activated. *GCLC* (the catalytic subunit of glutamate–cysteine ligase), the key rate-limiting enzyme converting L-glutamate to L-glutamylcysteine, showed pronounced expression heterogeneity, with significant up-regulation in the KAR-primed group, indicating regulatory variability across samples at the initial step of glutathione biosynthesis. In the conversion between glutathione (GSH) and oxidized glutathione (GSSG), four of the eight glutathione reductase (*GSR*) genes were significantly up-regulated in the KAR-primed group, while the other four were significantly down-regulated relative to CK2. The genes related to the downstream glutathione catabolism of GSSG were generally expressed at low levels (purple area), suggesting that the catabolic pathway may be suppressed (Figure 14).

In addition, in the NADPH-generating pathway associated with glutathione metabolism, most genes—including glucose-6-phosphate dehydrogenase (*G6PD*), 6-phosphogluconate dehydrogenase (*PGD*), and isocitrate dehydrogenase (*IDH*)—were significantly up-regulated in the KAR-treated group, indicating that KAR priming actively modulates NADPH supply within the glutathione metabolism pathway and thereby enhances glutathione biosynthesis (Figure 14).

Overall, gene expression along the glutathione metabolism pathway showed significantly differential regulation across samples, involving multiple steps including biosynthesis, reduction, and coenzyme supply, all of which may influence cellular redox status and antioxidant capacity.

#### 3.6.4. qRT-PCR Analysis

**Figure 15:**
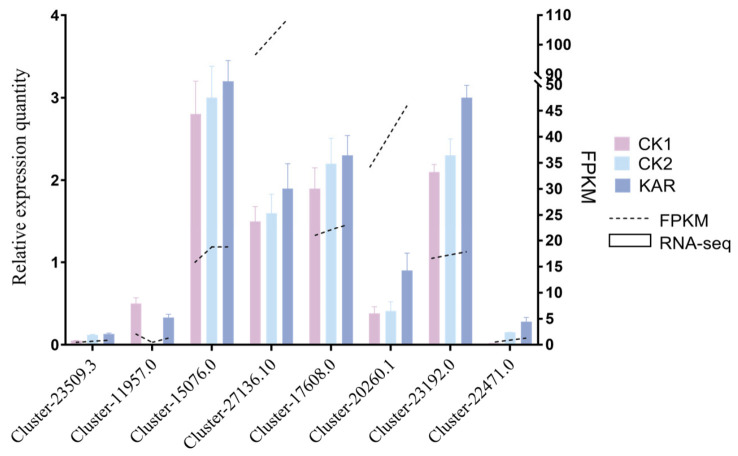
qRT-PCR validation of eight core genes.

At the same time, Real-time quantitative PCR (qRT-PCR) validation was performed on eight randomly selected DEGs. The expression trends matched the transcriptome sequencing data closely, confirming the reliability of the transcriptome data (Figure 15).

## 4. Discussion

### 4.1. Effects of KAR Priming on Germination and Physiological Characteristics of I. serra Seeds Under Heat Stress

Heat stress is a key environmental factor limiting the propagation of medicinal plants in warm regions. Extensive studies have shown that it disrupts cellular membrane integrity, drives the substantial accumulation of reactive oxygen species (ROS), and interferes with multiple key metabolic processes during seed germination [5,6]. Karrikin have attracted broad attention in recent years because they are active at sub-micromolar concentrations and act through the specific KAI2–MAX2–SMAX1 signaling pathway [15,18,30]. In this study, a systematic comparison of four priming agents at 35 °C showed that only 0.05 µmol/L KAR resulted in significantly higher germination than the water-primed control, achieving a final germination rate of 94.0%—about 15 percentage points higher than that of water priming. From the germination kinetic curves, the KAR-treated group reached 97.1% germination at 168 h, slightly higher than that of the 25 °C unstressed control, indicating that KAR improved *I. serra* germination under heat stress, a result similar to that in [31]. For seedling morphology, the hypocotyl length and cotyledon greenness of KAR-treated seedlings returned to levels close to those of the unstressed control (CK1), consistent with the finding in [32] that KAR promotes hypocotyl elongation and chlorophyll accumulation in *Arabidopsis* and *Brassicaceae* species. Finally, one limitation of our sampling design deserves mention. Because KAR treatment accelerated germination, collecting all samples at a fixed 54 h post-imbibition meant comparing seeds at different developmental stages—KAR-treated seeds were more advanced than those in CK2. As a result, our multi-omics data likely capture a blend of KAR-mediated thermotolerance and developmental shifts linked to germination progression. This developmental mismatch may obscure the specific molecular pathways involved in stress protection. Moving forward, synchronizing samples based on radicle emergence rather than clock time would help separate KAR’s growth-promoting effects from its role in heat mitigation.

In addition, under heat stress, *I. serra* membrane damage was significantly aggravated: MDA content rose by 44% compared with CK1, and REC rose by 24%, both reflecting heat-induced membrane lipid peroxidation. After KAR priming, MDA dropped back to 4.73 nmol g^−1^ FW, and REC fell significantly relative to CK2 to 37.7%, indicating that KAR priming effectively alleviates heat-induced oxidative damage to the seed membrane system, consistent with the results of [33].

SOD and CAT showed contrasting responses under KAR treatment. The most plausible interpretation is that KAR priming actually reduced the oxidative pressure imposed on the system, rather than uniformly up-regulating antioxidant enzymes. Under heat stress alone, the excessive ROS necessarily drove a compensatory induction of SOD; CAT, as a heme-containing enzyme, is sensitive to both thermal denaturation and product inhibition by H_2_O_2_, leading to a decline in activity. After KAR priming reduced the upstream oxidative load, the SOD compensatory response was able to retreat, while CAT activity was maintained at the control level or even higher. This interpretation aligns with [34,35], which observed that during seed dormancy release, KAR priming up-regulated the activity of SOD, CAT, glutathione reductase, and ascorbate peroxidase and suppressed the oxidative damage caused by excessive ROS accumulation. It is also consistent with the theoretical framework proposed by [36], in which the rebalancing of the antioxidant network, rather than the unidirectional up-regulation of enzyme activity, is the core of plant stress adaptation.

### 4.2. Effects of KAR Priming on Transcriptome and Metabolome of I. serra

Transcriptomics and metabolomics can systematically reveal plant responses to stress and exogenous treatments from two complementary perspectives—the gene expression and metabolite levels—and have become commonly used approaches for studying the molecular mechanisms of seed priming [37].

In this study, the CK2 vs. CK1 contrast induced the differential expression of 6026 genes, indicating extensive heat-induced effects on the transcriptional regulatory network of germinating *I. serra* seeds. The KEGG enrichment analysis of these heat-induced DEGs ranked protein processing in the endoplasmic reticulum, ribosome-related pathways, and several heat stress response pathways at the top; the heat shock protein family also displayed a typical induction pattern—of the 158 *HSPs* detected, 115 (72.8%) were up-regulated under heat stress alone, with the *HSP90* subfamily showing the most pronounced induction. After KAR priming, however, the number of DEGs dropped sharply: only 706 DEGs were identified in KAR vs. CK2, about one tenth of the heat-induced scale. The *HSP* family, which would normally be expected to be further up-regulated after KAR priming, instead showed an overall attenuation—126 of the 158 *HSPs* were down-regulated in KAR vs. CK2, contrary to most related reports [38,39,40]. One interpretation, therefore, is that KAR priming promotes seed germination not by reinforcing the heat shock cascade but by alleviating its upstream driving factors. Metabolomic analysis complemented the transcriptomic findings. The KAR vs. CK2 contrast identified 428 DAMs, dominated chemically by amino acids, organic acids, and lipids—a profile similar to that reported by [41].

### 4.3. KAR Priming Regulates the Glutathione Metabolism Pathway in I. serra

Glutathione (GSH), one of the most important nonenzymatic antioxidants in plants, plays an indispensable role in maintaining cellular redox homeostasis, detoxifying exogenous compounds, and defending against abiotic stress [42]. In this study, the transcriptomic, metabolomic, and physiological data converged on glutathione metabolism from multiple levels, indicating that this pathway is the core mechanism by which KAR priming alleviates the high-temperature germination barrier in *I. serra*.

In this study, key glutathione metabolism pathway genes including *GSS* (e.g., cluster-23192.0), *GSR* (e.g., cluster-27136.10), *OPLAH* (e.g., cluster-24751.0), and *G6PD* (e.g., cluster-20260.1) were significantly highly expressed in the KAR-primed group. Heat stress is a typical abiotic stress that disrupts the cellular redox balance, triggers extensive ROS bursts, and causes protein denaturation, membrane damage, and metabolic disorder, severely inhibiting normal plant growth and photosynthesis [43]. *GSS*, as the rate-limiting gene for glutathione biosynthesis, catalyzes the condensation of γ-glutamyl cysteine with glycine to form GSH, directly increasing endogenous GSH reserves under heat stress and providing the core substrate for the antioxidant detoxification system [44]. *GSR* continuously reduces heat-induced GSSG, regenerates functional GSH, stabilizes the intracellular GSH/GSSG redox balance, and effectively alleviates heat-induced oxidative damage [45]. *G6PD*, the rate-limiting enzyme of the pentose phosphate pathway, is significantly up-regulated under heat stress and continuously generates NADPH to supply reducing power for *GSR*-mediated GSH regeneration, sustaining the efficient operation of the glutathione antioxidant system and improving plant thermotolerance [46]. In addition, *OPLAH* participates in the regulation of lipid oxidation metabolism, alleviating membrane lipid peroxidation caused by heat stress and helping maintain the structural and functional stability of cellular membranes under high temperature [47]. Based on these results, KAR is proposed to enhance plant antioxidant capacity under heat stress by inducing high expression levels of the *GSS*, *GSR*, *G6PD*, and *OPLAH* gene series along multiple dimensions—GSH synthesis, redox balance maintenance, reducing power supply, and membrane repair—thereby efficiently scavenging heat-induced excess ROS and improving the thermotolerance and oxidative stress resistance of *I. serra* seeds. The metabolomic data closely matched the transcriptomic results. Five GSH-related metabolites showed an up-regulation trend after KAR treatment: GSH content was the lowest in CK2 and rose significantly after KAR treatment to approach normal levels, and γ-glutamylglutamate, S-lactoylglutathione, and other derivatives displayed the same pattern. These findings are consistent with previous reports [48].

The GSH pathway regulatory mechanism identified in this study is also consistent with conclusions from various plant stress studies. Research in crops such as *Pogostemon cablin* and tomato has confirmed that exogenous GSH can enhance plant stress tolerance and maintain redox homeostasis [41,49], supporting the role of the GSH pathway as the core route by which KAR alleviates heat stress in *I. serra*. GSH has also been reported to regulate the heat shock transcription factor-mediated stress response [50], which also explains the down-regulation of *HSP* genes observed after KAR treatment in this study: KAR priming may reduce the plant’s dependence on heat shock emergency protection by repairing GSH metabolic balance and lowering cellular oxidative pressure, ultimately enabling the effective recovery of seed germination under high temperature.

This study also has limitations. The multi-omics samples were collected uniformly at 54 h after imbibition, without aligning sampling to developmental stage. At this timepoint, approximately 50% of seeds in the KAR group had completed radicle protrusion, whereas the heat stress group had not yet entered this germination stage; therefore, part of the omics differences may stem from different germination progress rather than being solely attributable to KAR signal regulation. Future work could refine the regulatory mechanisms of KAR through molecular genetic experiments, multi-timepoint time-series sampling, and developmental-stage-aligned trials, combined with summer field validation experiments, in order to advance the application of this technology to large-scale *I. serra* propagation.

## 5. Conclusions

In this study, we used *I. serra* as the experimental material, and the optimal heat stress temperature was determined to be 35 °C through heat tolerance germination experiments. Among the priming treatments, 0.05 µmol/L KAR priming achieved the best effect, raising the final germination rate of *I. serra* seeds from 79% (water priming) to 94%; KAR priming also significantly improved all germination indices at high temperature. For physiological indicators, KAR priming significantly reduced MDA, relative membrane conductivity, and SOD levels in heat-stressed *I. serra* seeds; significantly increased CAT levels; and increased internal GSH levels. The multispectral phenotyping of 7-day-old seedlings showed that KAR priming differed from water priming at high temperature at specific wavelengths such as 880 and 970 nm; KAR-primed seedlings also had larger, fuller cotyledons, and nCDA visualization showed redder coloration and greater vigor in KAR-primed seedlings. Transcriptomic and metabolomic analyses indicated that KAR priming promotes *I. serra* seed germination under heat stress mainly by acting on the glutathione metabolism pathway. Integrated transcriptomic–metabolomic analysis further identified cluster-23509.3, cluster-15076.0, and cluster-22471.0 as key differentially expressed genes by which KAR priming alleviates heat stress and regulates glutathione biosynthesis, safeguarding normal seed germination. The findings provide a theoretical basis for the molecular mechanism of KAR priming under heat stress and offer new insights for the summer cultivation of *I. serra* in the Lingnan region of China.

## Data Availability

Since the multi-omics data comes from a Student thesis, to protect data privacy, we will not upload or publish the raw data from this study; however, if necessary, the data presented in this study can be obtained from the corresponding author upon request.

## Appendix A

**Table A1:** Germination indices of *I. serra* seeds at different temperatures.

Treatment	Germination Potential (%)	Germination Percentage (%)	Germination Index	Vigor Index	Root and Shoot Length (cm)
20 °C	68.00 ± 3.65 b	90.00 ± 2.94 b	33.70 ± 0.78 b	59.02 ± 1.82 b	1.75 ± 0.06 c
25 °C	83.00 ± 3.83 a	94.80 ± 0.96 a	38.89 ± 0.90 a	86.8 ± 1.97 a	2.23 ± 0.03 a
30 °C	64.50 ± 4.80 b	85.00 ± 0.82 c	32.33 ± 1.16 c	58.94 ± 2.19 c	1.83 ± 0.07 b
35 °C	60.80 ± 2.30 c	72.30 ± 1.32 d	24.74 ± 1.59 d	16.71 ± 2.71 d	0.68 ± 0.11 d

Note: All data are presented as “mean ± standard deviation”; significance analysis was conducted using Duncan’s multiple range test, with different letters indicating significant differences (*p* < 0.05); *n* = 3.

**Table A2:** Germination indices of *I. serra* seeds induced by KAR initiators.

Treatment	Germination Potential (%)	Germination Percentage (%)	Germination Index	Vigor Index	Root and Shoot Length (cm)
CK1	58.80 ± 2.30 c	70.50 ± 2.20 c	21.64 ± 1.61 d	14.51 ± 2.31 c	0.62 ± 0.11 b
CK2	69.80 ± 2.30 b	79.30 ± 1.32 b	29.76 ± 1.57 b	19.77 ± 1.09 b	0.68 ± 0.02 b
0.05 µmol/L	86.00 ± 1.95 a	94.00 ± 1.94 a	35.96 ± 1.99 a	31.73 ± 2.05 a	0.88 ± 0.01 a
0.1 µmol/L	68.80 ± 1.76 b	84.00 ± 1.51 b	32.94 ± 0.98 ab	20.65 ± 1.95 b	0.71 ± 0.04 b
0.2 µmol/L	60.25 ± 1.35 c	80.00 ± 1.83 b	29.29 ± 1.61 c	22.06 ± 0.33 b	0.67 ± 0.02 b

Note: All data are presented as “mean ± standard deviation”; significance analysis was conducted using Duncan’s multiple range test, with different letters indicating significant differences (*p* < 0.05); *n* = 3.

**Table A3:** Transcriptomic data quality control analysis.

Sample	Raw Reads	Clean Reads	Clean Base (G)	Error Rate (%)	Q20 (%)	Q30 (%)	GC Content (%)
CK1-1	50,795,790	49,767,998	7.47	0.01	98.41	95.20	48.20
CK1-2	62,432,294	61,395,758	9.21	0.01	98.29	94.86	47.83
CK1-3	55,484,346	54,375,406	8.16	0.01	97.73	93.21	47.87
CK2-1	50,118,750	49,332,264	7.40	0.01	98.06	94.11	47.42
CK2-2	52,027,222	51,123,038	7.67	0.01	98.12	94.20	47.62
CK2-3	56,380,492	55,570,660	8.34	0.01	98.19	94.47	47.52
KAR-1	56,731,134	55,799,550	8.37	0.01	98.14	94.30	47.33
KAR-2	49,817,998	48,945,580	7.34	0.01	98.11	94.22	47.49
KAR-3	59,401,886	58,405,956	8.76	0.01	98.25	94.63	47.32
